# Pain and patient‐reported experience after freehand transperineal prostate biopsy under local anaesthesia

**DOI:** 10.1002/bco2.70290

**Published:** 2026-10-04

**Authors:** Keu Maoate, Josh Tiro, Ali Hooshyari, Maria McCoy, Christian Kirk, Lodewikus Vermeulen, Amir Zarrabi, Luiz Gustavo Modelli de Andrade, Paulo Roberto Kawano, Flavio Vasconcelos Ordones

**Affiliations:** ^1^ Department of Urology Tauranga Public Hospital Tauranga New Zealand; ^2^ Faculty of Health and Medical Sciences University of Auckland Auckland New Zealand; ^3^ Nephrology Division São Paulo State University (UNESP) Botucatu Brazil; ^4^ Urology Division São Paulo State University (UNESP) Botucatu Brazil

**Keywords:** cohort study, local anaesthesia, pain, patient‐reported outcome measures, prostate biopsy, transperineal

## Abstract

**Objectives:**

To characterise patient‐reported pain, anxiety, satisfaction and willingness to undergo a repeat procedure, along with complication rate and diagnostic yield, in a large consecutive single‐centre cohort of freehand transperineal prostate biopsies (TPPB) performed under local anaesthesia (LA) alone.

**Patients and methods:**

Consecutive freehand TPPB cases entered into a single‐centre clinical registry between 27 April 2022 and 12 January 2026 were reviewed. Cases performed under general anaesthesia or under LA with intravenous sedation were excluded. The primary outcome was patient‐reported overall procedural pain on a 0–10 cm visual analogue scale (VAS), completed immediately after the procedure. Secondary outcomes were VAS scores for anxiety, distress, physical needs, emotional needs and satisfaction; association between pain and anxiety; procedural failure; complication rate; and detection of clinically significant prostate cancer (csPCa, ISUP grade group ≥2). The tertiary outcome was willingness to undergo a repeat biopsy if clinically indicated.

**Results:**

Of 800 consecutive cases, 777 (97.1%) performed under LA alone formed the analytic cohort. Median overall procedural pain was 2.0 (IQR 1.0–3.0), and severe pain (VAS ≥ 7) was reported by 45 of 760 patients (5.9%). Anxiety was moderately correlated with pain (Spearman ρ = 0.51, 95% CI 0.44–0.57), and severe pain was ten times more common above than below the median anxiety score (10.8% vs. 1.0%). Overall, 748 of 756 patients (98.9%, 95% CI 97.9–99.5%) would undergo the procedure again if clinically indicated, including 42 of the 45 (93%) who reported severe pain. Procedural failure occurred in 5 of 777 cases (0.6%) and a complication was noted in 26 of 776 (3.4%). CsPCa was identified in 477 of 769 patients (62.0%), rising stepwise with PI‐RADS score (1–2: 18.3%; 3: 22.6%; 4: 70.0%; 5: 88.4%).

**Conclusions:**

Freehand TPPB under LA alone is well tolerated in the outpatient setting. Pain is low overall, severe pain is uncommon, and almost all patients would undergo the procedure again as indicated. Patient‐reported anxiety is moderately associated with procedural pain and is a plausible modifiable target.

## 1 INTRODUCTION

Prostate cancer is one of the most commonly diagnosed cancers in men worldwide.^1^, ^2^ Although feasibility studies suggest that, in highly selected patients with concordant high‐suspicion multiparametric MRI (mpMRI) and prostate‐specific membrane antigen positron emission tomography, radical treatment may be planned without prior histological confirmation,^3^, ^4^ the authors of those series emphasise that a biopsy‐free pathway is not current standard practice.^3^ Prostate biopsy therefore remains the routine method of confirming the diagnosis.

The transperineal (TP) route is now the preferred approach and is recommended as first choice in the European Association of Urology (EAU) guidelines,^5^ with the American Urological Association/Society of Urologic Oncology dedicating a full section of its 2023 early‐detection update to biopsy technique.^6^ The principal advantages of the TP route are more complete sampling of the gland, particularly the anterior zone,^7^ and a markedly lower risk of post‐biopsy sepsis: A systematic review and meta‐analysis of ten randomised trials (4188 biopsies) reported a 77% reduction in the odds of hospitalisation for infection compared with the transrectal route (OR 0.23, 95% CI 0.10–0.54).^8^ The randomised NORAPP trial (*n* = 555) further showed that omitting antibiotic prophylaxis before TP biopsy under local anaesthesia did not increase infection rates,^9^ a position now endorsed in the EAU guidelines.^10^ A systematic review and meta‐analysis of TPPB series reached the same conclusion, reporting a low risk of infectious complications both with or without antibiotic prophylaxis.^11^ In parallel, mpMRI has become integral to the diagnostic pathway following the PRECISION trial, in which an MRI‐directed pathway detected clinically significant cancer in 38% of biopsy‐naive men compared with 26% for standard transrectal ultrasound‐guided biopsy while allowing 28% of men with a non‐suspicious MRI to avoid biopsy altogether.^12^ TP biopsy is now regularly performed under LA alone in the outpatient clinic setting, with feasibility demonstrated in multicentre cohorts.^13^, ^14^, ^15^

The remaining concern for TP biopsy under LA is patient experience, in particular procedural pain. The TRANSLATE randomised trial (*n* = 1126) confirmed that LA‐TP biopsy detected more clinically significant cancer than transrectal biopsy (60% vs. 54%; OR 1.32, 95% CI 1.03–1.70; *p* = 0.031) but also that participants more commonly described the TP procedure as immediately painful and embarrassing (38% vs. 27%; OR 1.84, 95% CI 1.40–2.43).^16^ The PREVENT trial (*n* = 658) similarly found worse periprocedural pain in the TP arm, although the absolute difference was small and had resolved by 7 days.^17^ Existing research of TP biopsy outcomes has treated pain as one of several secondary endpoints alongside diagnostic yield and complications,^18^, ^19^ and dedicated pain meta‐analyses remain restricted to the transrectal route.^20^, ^21^ The largest dedicated pain datasets to date are the 500‐patient T‐PAIN study, in which the median overall VAS was 2 (IQR 0–3),^22^ and the multicentre study of 1008 men reported by Marra et al., in which pain was the primary endpoint and severe anxiety, younger age and longer procedure time were independent predictors of severe biopsy pain.^23^

Data from large single‐centre series performing freehand TP biopsy under LA alone remain limited yet are needed to benchmark the literature against practice—particularly now that the cost‐effectiveness of LA delivery in the outpatient clinic has been formally evaluated.^24^, ^25^ We report the pain and patient‐reported outcome experience of 777 consecutive freehand TPPB performed under LA at a single centre between 2022 and 2026, with the aim of characterising the magnitude and distribution of procedural pain, the rate of severe pain (VAS ≥ 7), the association between patient‐reported anxiety and pain, willingness to undergo a repeat biopsy, complications captured within 30 days of biopsy and the diagnostic yield for clinically significant prostate cancer by PI‐RADS category.

## 2 METHODS

### 2.1 Study design and setting

This was a consecutive, single‐centre observational cohort study of all freehand TPPB performed under LA between 27 April 2022 and 12 January 2026 at Tauranga Public Hospital, a secondary urology service in Tauranga, Bay of Plenty, New Zealand. Cases were entered prospectively into a departmental clinical registry.

The study is reported in accordance with the STROBE statement for observational studies. Ethical approval was granted by the New Zealand Health and Disability Ethics Committee (reference 2022‐206) and by the local Māori research review committee. Written informed consent was obtained from all patients. All research was performed in accordance with relevant guidelines and regulations, including the Declaration of Helsinki.

### 2.2 Participants

All consecutive adult men (≥18 years) undergoing freehand TPPB at the participating centre during the study period were eligible. Indications were: suspected prostate cancer (raised PSA, abnormal digital rectal examination or a suspicious lesion on mpMRI) or surveillance biopsy in men on active surveillance. All patients underwent pre‐biopsy mpMRI. There were no exclusions on the basis of prior biopsy status, prostate volume, comorbidity or PSA level. Cases performed under general anaesthesia (*n* = 2) or under local anaesthesia with intravenous sedation (*n* = 20), and one case in which the anaesthetic regimen was not recorded, were excluded, leaving 777 cases performed under local anaesthesia alone as the analytic cohort (Figure 1).

**FIGURE 1:**
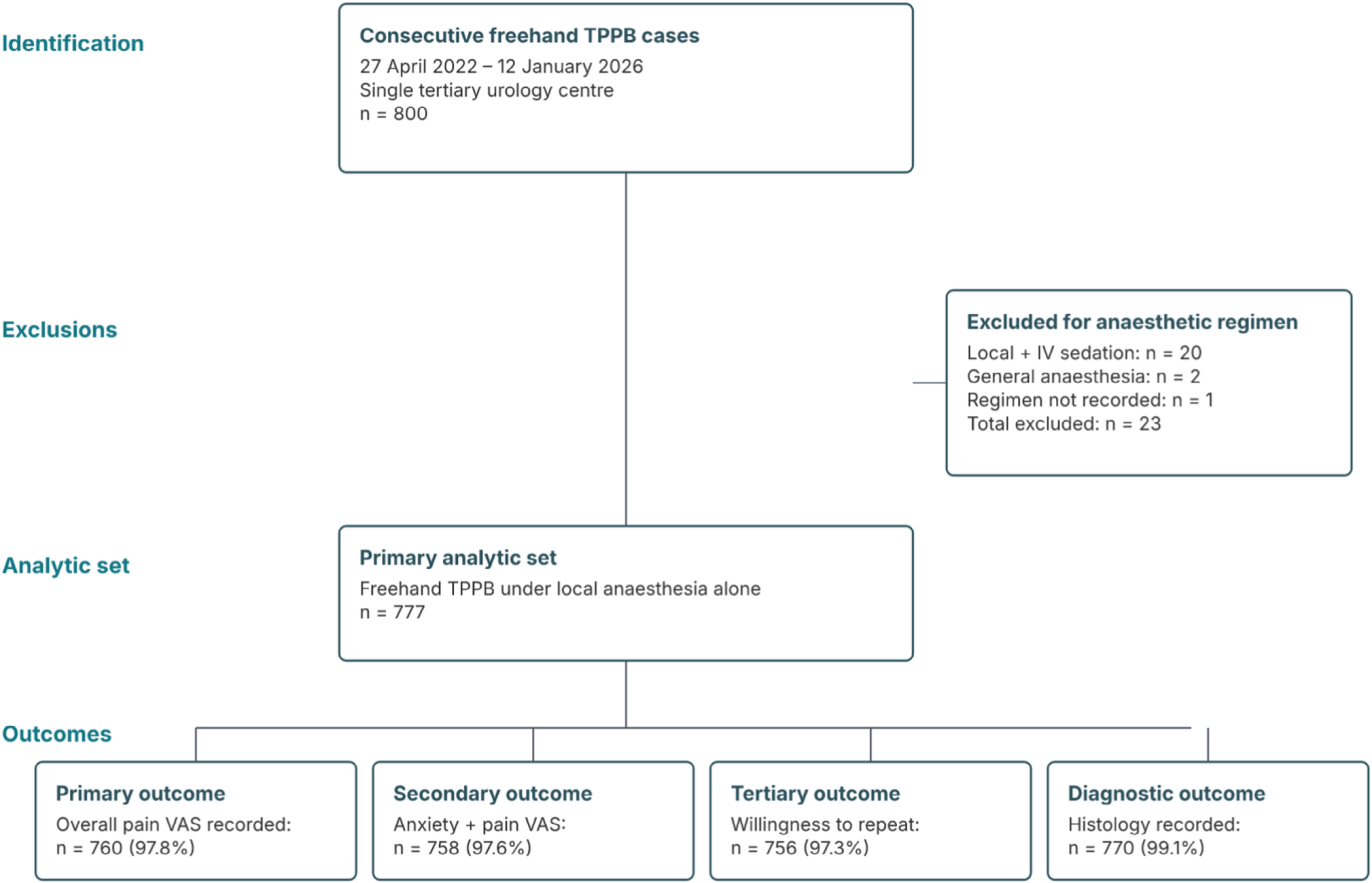
STROBE flow diagram for the freehand TPPB cohort. Of 800 consecutive cases performed between 27 April 2022 and 12 January 2026, 23 were excluded on the basis of anaesthetic regimen (20 local anaesthesia plus intravenous sedation, two general anaesthesia, one regimen not recorded), leaving 777 cases performed under local anaesthesia alone as the analytic cohort. Outcome‐specific denominators are shown for the pain, anxiety, willingness‐to‐repeat and histology analyses.

### 2.3 Biopsy procedure

All biopsies were performed using a freehand TP technique with ultrasound guidance and the PrecisionPoint Transperineal Access System.^26^ Procedures were performed in an outpatient urology clinic or, where clinically indicated, in a day‐case theatre setting. Two consultant urologists performed 99.4% of all procedures, with the remaining cases performed by other clinicians under their direct supervision.

The local anaesthetic regimen was delivered in three steps as previously described by Ordones et al.^27^

1. The use of cold spray (ethyl chloride) on the skin prior to subcutaneous block.
2. Bilateral skin and subcutaneous block with 1% lignocaine (10 ml) and sodium bicarbonate 8.4% (1 ml) using a 21‐G needle. 5 ml was placed either side of the midline.
3. Bilateral pelvic wall muscle block, followed by periapical triangle block using a 20‐G spinal needle, and 8 ml of the solution (lignocaine 1% and sodium bicarbonate) on each side. A transrectal ultrasound probe and the PrecisionPoint device are used.

The biopsy was performed 3–5 min after administration of local anaesthesia. The same regimen was used in all 777 included cases. Sampling strategy was determined by the operator based on the pre‐biopsy mpMRI: combined targeted plus systematic sampling for most patients (75.7%), systematic sampling alone where no MRI target was identified (19.8%) or targeted sampling alone in selected cases (4.4%). Systematic biopsies were performed using a modified Ginsburg protocol. The number of cores taken was at the operator's discretion (median 18, interquartile range 16–23). All biopsies were performed freehand using the Precision Point device, with cognitive fusion and no software assistance.

### 2.4 Variables and data sources

Variables were captured at the time of the procedure and entered into the departmental registry by the operating clinician. Pre‐procedure variables were age, body mass index, ethnicity, PSA, prostate volume, PSA density, biopsy history, prior active‐surveillance status, mpMRI status and the highest PI‐RADS v2.1 lesion score. Intra‐procedure variables were setting (outpatient clinic vs. day‐case theatre), operating clinician, total number of cores, sampling strategy (targeted only, systematic only or combined) and total procedure time. The total procedure time was recorded by the operating clinician at the time of the procedure and was defined as the interval from the initial administration of local anaesthetic to the removal of the transrectal ultrasound probe.

The primary outcome was patient‐reported overall procedural pain on a 0–10 cm visual analogue scale (VAS; 0 = *no pain*, 10 = *worst pain imaginable*), self‐completed by the patient immediately after the procedure using the institutional patient‐experience questionnaire.

Secondary patient‐reported outcomes were VAS scores for procedural anxiety, distress, perceived physical needs, perceived emotional needs and overall satisfaction, together with a binary willingness‐to‐repeat question. Pain at later timepoints was not captured. Other secondary outcomes were procedural failure (the procedure aborted before completion) and complication rate. The diagnostic outcome was the highest ISUP grade group on biopsy histology, with clinically significant prostate cancer (csPCa) defined as ISUP grade group ≥2. Complications were recorded within 30 days of biopsy.

For interpretation, VAS scores were grouped into conventional severity bands: 0 = *none*, 1–3 = *mild*, 4–6 = *moderate* and 7–10 = *severe*. Severe pain and severe anxiety were accordingly defined as a VAS of 7 or above.

### 2.5 Statistical analysis

Continuous variables are presented as median (interquartile range) and, where informative, mean (standard deviation); categorical variables as *n* (%). Missing data were not imputed, and every variable is reported with its observed denominator.

The primary outcome was the distribution of the overall procedural pain VAS in the analytic cohort, summarised as the median, IQR, mean, standard deviation and the proportion of patients reporting severe pain (VAS ≥ 7). The anxiety–pain association was assessed with Spearman's rank correlation (two‐sided), with a 95% confidence interval obtained from 10 000 bootstrap resamples. The willingness‐to‐repeat outcome was reported as a proportion with an exact binomial 95% confidence interval; the ‘yes’ and ‘no’ subgroups were compared using the Mann–Whitney *U* test for continuous variables and Fisher's exact test for categorical variables (procedural failure, any complication, severe pain). Because only eight patients answered ‘no’, these subgroup comparisons are descriptive, and no multivariable model was fitted. Consistent with the descriptive aim of this report, multivariable modelling of the primary outcome was not pre‐specified and is not presented; all reported associations are unadjusted. All tests were two‐sided and *p* < 0.05 was considered statistically significant.

Six patients whose histology was recorded with a non‐standard code (five atypical small acinar proliferation and one uninterpretable entry) and two with no histology recorded could not be assigned an ISUP grade group and were excluded, giving 769 patients with evaluable histology. Analyses were performed in R version 4.4.1 (R Foundation for Statistical Computing, Vienna, Austria).

## 3 RESULTS

### 3.1 Participants

Between 27 April 2022 and 12 January 2026, 800 consecutive freehand TPPB were performed and entered into the clinical registry. Twenty‐three cases were excluded on the basis of anaesthetic regimen: 20 performed under local anaesthesia with intravenous sedation, two under general anaesthesia, and one in which the regimen was not recorded. A total of 777 cases performed under LA alone form the analytic cohort (Figure 1).

### 3.2 Cohort characteristics

Baseline characteristics, procedure details and outcomes are summarised in Table 1. Median age was 67 years and median BMI 28.0 kg/m^2^. The cohort was predominantly of European ethnicity (77.9%), with significant New Zealand Māori representation (19.4%). Median pre‐biopsy PSA was 8.0 ng/ml and median prostate volume 42 ml. All patients underwent pre‐biopsy mpMRI. The highest PI‐RADS lesion score was 4 in 321 patients (41.3%) and 5 in 244 (31.4%), with 206 patients (26.5%) scoring 1–3 and four (0.5%) with no score recorded.

**TABLE 1:** Baseline characteristics, procedure details and outcomes of 777 consecutive freehand transperineal prostate biopsies performed under local anaesthesia alone.

Characteristic	Value
Patient characteristics	
Age, years—median (IQR)	67 (62–71)
Age, years—mean (SD)	66.2 (7.5)
BMI, kg/m^2^—median (IQR); *n* = 685	28.0 (26.0–31.0)
Ethnicity	
European	605 (77.9%)
New Zealand Māori	151 (19.4%)
Asian	11 (1.4%)
Pasifika	5 (0.6%)
Other	5 (0.6%)
Clinical and imaging characteristics	
PSA, ng/mL—median (IQR)	8.0 (5.5–12.0)
Prostate volume, mL—median (IQR); *n* = 774	42 (32–58)
PSA density, ng/mL/cc—median (IQR); *n* = 774^a^	0.19 (0.13–0.29)
Pre‐biopsy mpMRI performed^b^	777 (100%)
Highest PI‐RADS lesion score	
1	59 (7.6%)
2	84 (10.8%)
3	63 (8.1%)
4	321 (41.3%)
5	244 (31.4%)
No discrete lesion (score 0)	2 (0.3%)
Not recorded	4 (0.5%)
Prior biopsy	
None	597 (76.8%)
Prior transperineal biopsy	87 (11.2%)
Prior transrectal biopsy	76 (9.8%)
Prior HoLEP/TURP	9 (1.2%)
Not recorded	8 (1.0%)
On active surveillance before biopsy	121 (15.6%)
Days from booking visit to biopsy—median (IQR); *n* = 738	62 (42–85)
Procedure details	
Setting	
Outpatient clinic	674 (86.7%)
Day‐case theatre	101 (13.0%)
Not recorded	2 (0.3%)
Anaesthesia—local anaesthesia alone	777 (100%)
Operator	
Operator A	291 (37.5%)
Operator B	481 (61.9%)
Other	5 (0.6%)
Sampling strategy	
Combined targeted + systematic	588 (75.7%)
Systematic only	154 (19.8%)
Targeted only	34 (4.4%)
Not recorded	1 (0.1%)
Number of cores—median (IQR); *n* = 769	18 (16–23)
Procedure time, min—median (IQR); *n* = 675	10 (9–12)
Procedural and diagnostic outcomes	
Procedural failure (aborted before completion)	5 (0.6%)
Complications (within first 30 days); *n* = 776	
None	750 (96.6%)
Vasovagal episode	14 (1.8%)
Infection (UTI)	8 (1.0%)
Bleeding	4 (0.5%)
Urinary retention	0 (0%)
Any complication	26 (3.4%)
Histology (ISUP grade group); *n* = 769^c^	
Benign	159 (20.7%)
ISUP 1	133 (17.3%)
ISUP 2	197 (25.6%)
ISUP 3	132 (17.2%)
ISUP 4	86 (11.2%)
ISUP 5	62 (8.1%)
Clinically significant prostate cancer (ISUP ≥2)	477/769 (62.0%)
Patient‐reported outcomes (post‐procedure VAS, 0–10 cm)	
Overall procedural pain—median (IQR); *n* = 760	2.0 (1.0–3.0)
Overall procedural pain—mean (SD)	2.4 (2.1)
Severe pain (VAS ≥ 7)	45/760 (5.9%)
Anxiety—median (IQR); *n* = 759	2.5 (1.0–5.0)
Severe anxiety (VAS ≥ 7)	138/759 (18.2%)
Distress—median (IQR); *n* = 753	1.0 (0.0–2.0)
Physical needs—median (IQR); *n* = 756	0.0 (0.0–1.0)
Emotional needs—median (IQR); *n* = 761	0.0 (0.0–1.0)
Satisfaction—median (IQR); *n* = 760^d^	0.0 (0.0–1.0)
Would undergo a repeat biopsy if indicated; *n* = 756	748 (98.9%)

*Note*: Values are *n* (%), median (IQR) or mean (SD) as indicated. Where data were not captured for the whole cohort, the observed denominator is shown.

^a^ PSA density = PSA/prostate volume.

^b^ On clinical review with the operating team, all 777 patients had a pre‐biopsy mpMRI; the source MRI field was recoded to correct 10 data‐entry errors.

^c^ Six patients whose histology carried a non‐standard code (five ‘ASAP’, one uninterpretable) and two with no histology recorded could not be assigned an ISUP grade group and are excluded from this denominator.

^d^ Satisfaction was rated on the same 0–10 scale, on which 0 denotes complete satisfaction and 10 complete dissatisfaction.

Most patients had no prior prostate biopsy (76.8%); 11.2% had undergone a previous TPPB, and 9.8% a previous TR biopsy. A total of 121 patients (15.6%) were on active surveillance before the index biopsy. Median time from booking visit to biopsy was 62 days (IQR 42–85), noting this includes patients on active surveillance who had scheduled biopsies arranged at varied intervals from the scheduled date.

Biopsies were performed in the outpatient urology clinic in 674 cases (86.7%) and in a day‐case theatre in 101 (13.0%). Two operators performed 99.4% of cases (37.5% and 61.9%, respectively). The dominant sampling strategy was combined targeted plus systematic biopsy (75.7%), with systematic only in 19.8% and targeted only in 4.4%. The median number of cores was 18, and the median procedure time was 10 min.

### 3.3 Primary outcome: Overall procedural pain

The overall procedural pain VAS was completed by 760 of the 777 patients (97.8%). Median pain score was 2.0 (Figure 2). 444 patients (58.4%) reported pain of 2.0 or less, and severe pain (VAS ≥ 7) was reported by 45 patients (5.9%).

**FIGURE 2:**
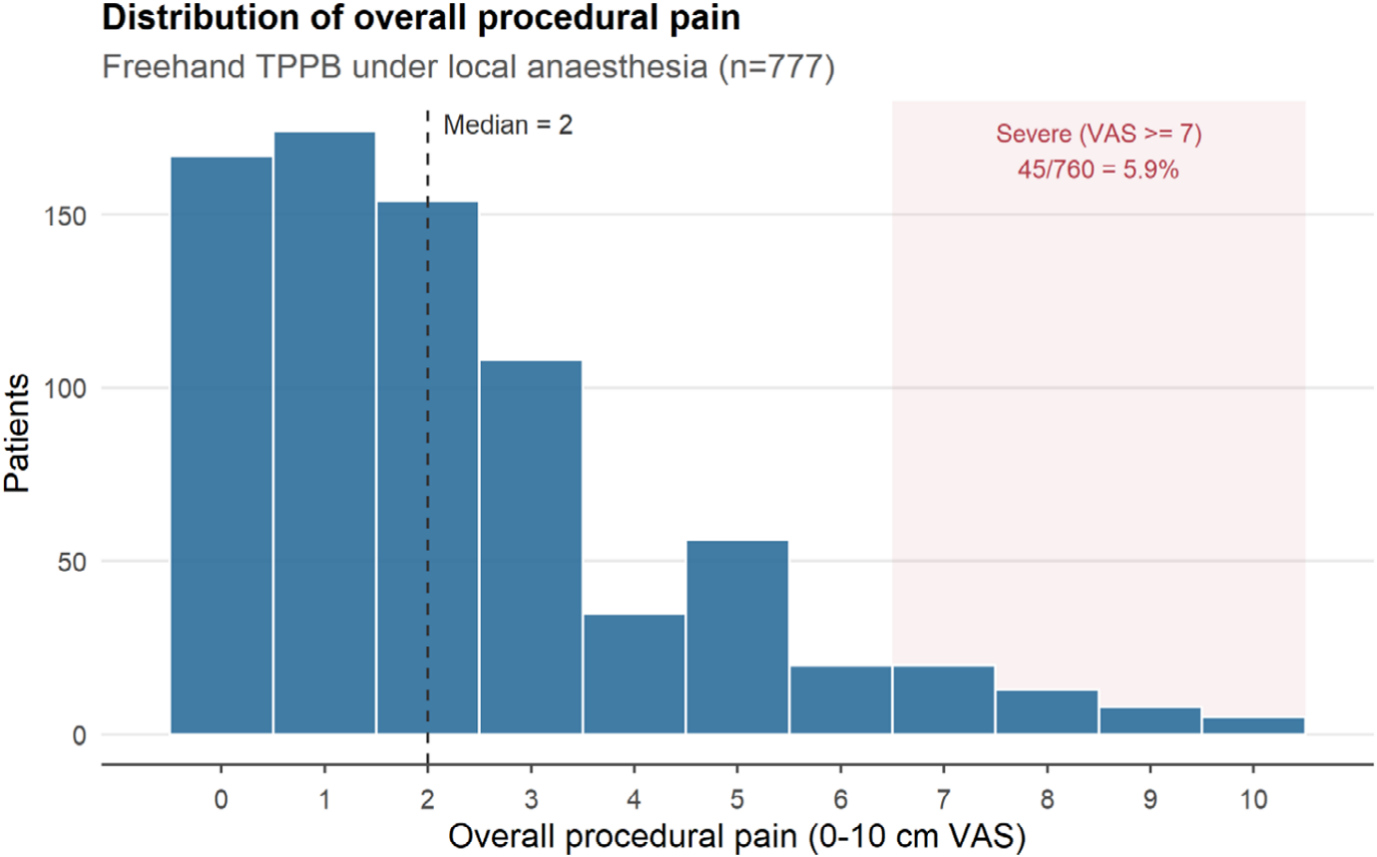
Distribution of patient‐reported overall procedural pain on a 0–10 cm VAS in the 760 patients with a recorded score. The distribution is right‐skewed, with a median of 2.0 (IQR 1.0–3.0); 5.9% of patients reported severe pain (VAS ≥ 7).

### 3.4 Secondary outcomes

#### 3.4.1 Anxiety and the anxiety–pain association

Procedural anxiety was reported by 759 patients (97.7%), with a median of 2.5; 138 patients (18.2%) reported severe anxiety (VAS ≥ 7). Among the 758 patients who completed both the anxiety and the pain VAS, the two were moderately positively correlated (Spearman ρ = 0.51, 95% bootstrap CI 0.44–0.57; *p* < 0.001) (Figure 3B). Mean pain rose across anxiety quartiles, from 1.0 in the lowest quartile to 3.7 in the highest. Severe pain was reported by 4 of 387 patients (1.0%) with anxiety at or below the cohort median (2.5) and by 40 of 371 (10.8%) with anxiety above the median—a 10‐fold difference. One patient who reported severe pain did not complete the anxiety VAS.

**FIGURE 3:**
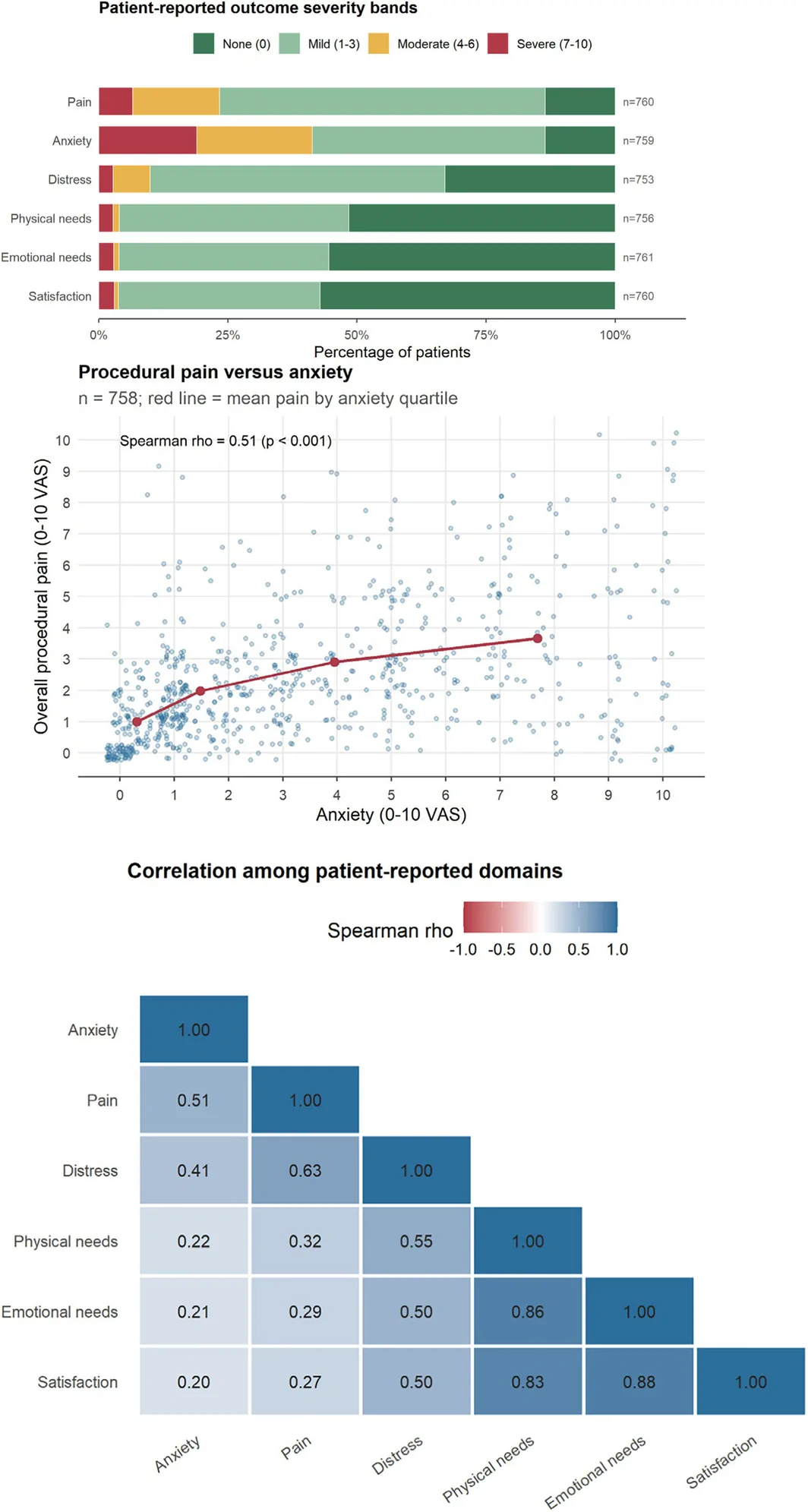
(A) Percentage of patients in each severity band across six VAS domains. Severity bands for six 0–10 cm VAS domains—overall pain, anxiety, distress, physical needs, emotional needs and satisfaction. (B) Overall procedural pain versus anxiety (*n* = 758); the connecting line shows mean pain within each anxiety quartile (spearman ρ = 0.51, *p* < 0.001). (C) Spearman correlation heatmap among the six patient‐reported outcome domains. Cells for which the correlation did not reach statistical significance (*p* ≥ 0.05) are left blank.

Both VAS scores were captured on the same post‐procedure questionnaire, so this association is cross‐sectional rather than predictive (see Discussion).

#### 3.4.2 Other patient‐reported outcomes

Distress, perceived physical needs and perceived emotional needs each had a median of 1.0 or lower, with distributions concentrated at the favourable end of the scale, as did patient‐reported satisfaction (median 0.0, IQR 0.0–1.0) (Figure 3A). The six patient‐reported domains were positively inter‐correlated, most strongly between distress and anxiety and between distress and pain (Figure 3C).

### 3.5 Tertiary outcome: Willingness to undergo a repeat biopsy

Of the 777 patients, 756 (97.3%) answered the willingness‐to‐repeat question. Of these, 748 (98.9%) reported that they would undergo the procedure again if clinically indicated; eight (1.1%) reported that they would not.

Compared with those willing to repeat, the eight patients who were unwilling reported higher pain (median 4.5 vs. 2.0, *p* = 0.017), higher anxiety (6.5 vs. 2.5, *p* = 0.012), higher distress (3.5 vs. 1.0, *p* = 0.007) and lower satisfaction (2.0 vs. 0.0, *p* = 0.009) (Mann–Whitney *U*). Severe pain was reported by three of the eight (37.5%) compared with 5.6% of those willing to repeat (Fisher's exact *p* = 0.009), and two of the eight (25.0%) had a procedural failure compared with 0.4% of those willing to repeat (*p* = 0.001). Age, prostate volume, PSA, number of cores and the overall complication rate did not differ between the groups.

Of note, 42 of the 45 patients (93%) who reported severe procedural pain nonetheless indicated that they would undergo a repeat biopsy if clinically indicated. Severe pain alone therefore does not appear to be the dominant driver of refusal in this cohort; rather, it is the combination of pain with a procedure that could not be completed.

### 3.6 Procedural outcomes and complications

Procedural failure occurred in five of 777 cases (0.6%). A reason was documented in two of these (pain and inability to tolerate pain together with anxiety) and was not recorded in the remaining three. Within 30 days, 750 of 776 (96.6%) patients had no complication; there were 14 vasovagal episodes (1.8%), eight infections (1.0%) and four bleeding events (0.5%), giving an overall complication rate of 26 of 776 (3.4%). No cases of urinary retention were recorded.

### 3.7 Diagnostic yield and histology by PI‐RADS

Of the 769 patients with available histology, 477 (62.0%) had clinically significant prostate cancer (ISUP grade group ≥2). The overall risk‐group distribution was 20.7% benign, 17.3% low risk (ISUP 1), 42.8% intermediate risk (ISUP 2–3) and 19.2% high risk (ISUP 4–5).

Histology stratified by the highest pre‐biopsy PI‐RADS lesion score is shown in Table 2 and Figure 4. The csPCa detection rate rose stepwise with PI‐RADS score: 18.3% (26/142) at PI‐RADS 1–2, 22.6% (14/62) at PI‐RADS 3, 70.0% (222/317) at PI‐RADS 4 and 88.4% (214/242) at PI‐RADS 5. The composition of risk groups shifted correspondingly, with high‐risk disease (ISUP 4–5) accounting for 2.8% of biopsies at PI‐RADS 1–2 and 38.4% at PI‐RADS 5.

**TABLE 2:** Biopsy histology by highest pre‐biopsy PI‐RADS lesion score.

Highest PI‐RADS	Benign	Low (ISUP 1)	Intermediate (ISUP 2–3)	High (ISUP 4–5)	csPCa (ISUP ≥2)	n
PI‐RADS 1–2	58.5%	23.2%	15.5%	2.8%	**18.3%**	142
PI‐RADS 3	56.5%	21.0%	21.0%	1.6%	**22.6%**	62
PI‐RADS 4	9.8%	20.2%	54.3%	15.8%	**70.0%**	317
PI‐RADS 5	2.9%	8.7%	50.0%	38.4%	**88.4%**	242
**Total**	**20.4%**	**17.2%**	**43.0%**	**19.4%**	**62.4%**	**763**

*Note*: Restricted to the 763 patients with both an evaluable ISUP grade group and a PI‐RADS score of 1–5. Risk groups are derived from biopsy histology alone: benign (no cancer), low risk (ISUP 1), intermediate risk (ISUP 2–3) and high risk (ISUP 4–5). csPCa is defined as ISUP grade group ≥2. Percentages are row percentages within each PI‐RADS stratum.

**FIGURE 4:**
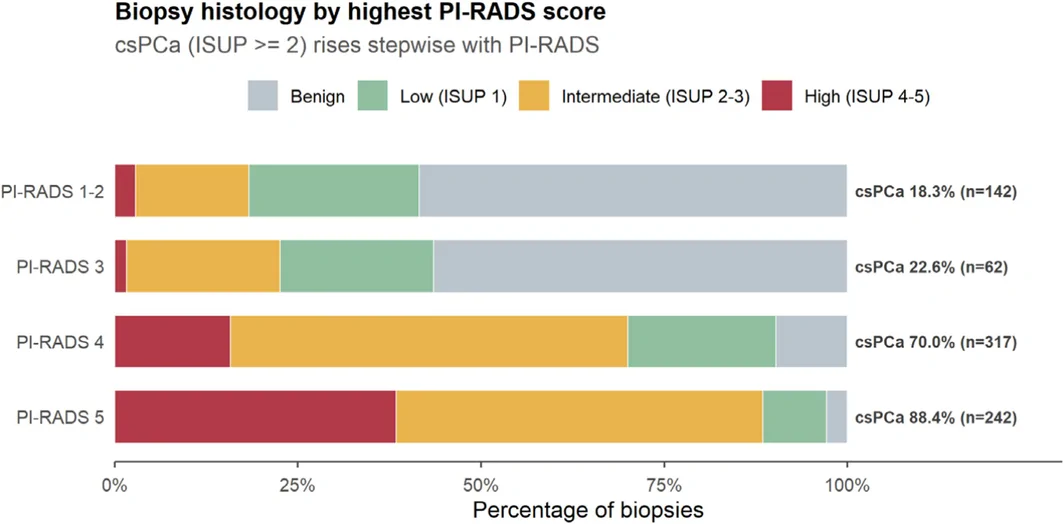
Composition of biopsy histology risk group by highest pre‐biopsy PI‐RADS lesion score. Detection of clinically significant prostate cancer (ISUP ≥2) rises stepwise from 18.3% at PI‐RADS 1–2 to 88.4% at PI‐RADS 5.

## 4 DISCUSSION

### 4.1 Principal findings

In 777 consecutive freehand TPPB performed under LA alone, patient‐reported procedural pain was low (median VAS 2.0, IQR 1.0–3.0), severe pain was uncommon (5.9%), procedural failure was rare (0.6%), and clinically significant prostate cancer was detected in 62.0% of patients with available histology. Almost all patients (98.9%) said they would undergo the procedure again if clinically indicated. Patient‐reported anxiety was moderately correlated with procedural pain (Spearman ρ = 0.51), and the small group unwilling to repeat the biopsy was distinguished by higher pain, anxiety and distress and by procedural failure, rather than by demographic or oncological factors.

### 4.2 Literature comparison

The largest dedicated pain dataset in this setting is the multicentre series of 1008 men reported by Marra et al., in which pain was the primary endpoint.^23^ Using a 0–10 numeric rating scale, they recorded mean pain of 3.9 (SD 2.1) during administration of LA and 3.1 (SD 2.3) during sampling, and on multivariable analysis severe anxiety (OR 2.99, 95% CI 1.83–4.89), younger age and longer procedure time were independently associated with severe biopsy pain. Our median VAS of 2.0 is lower than their mean score during sampling, although the scales, timing of assessment and access devices differ. The more important point of agreement is anxiety: Their strongest predictor of severe pain is reproduced in our cohort, in which severe pain was ten times more common in men with above‐median anxiety.

Our findings align with those summarised by Mac Curtain et al. in which pain and complications were reported as secondary endpoints alongside cancer detection^19^ and by the earlier systematic review of Kanagarajah et al.^18^ Our cohort adds one of the largest single‐centre datasets in which pain was the primary outcome and extends it with patient‐experience VAS scores across five further domains and with willingness to repeat.

The randomised trial of Waisman Malaret et al. provides comparison of post‐procedure PROMs between the TR and TP routes, reporting higher rates of pain and urinary dysfunction with the TP approach^28^; TRANSLATE reported higher rates of pain and embarrassment with TP biopsy.^16^ Although these studies show that the TP route may be more painful than transrectal, our cohort shows that the absolute level of pain in routine LA practice remains low and that 98.9% of patients would still undergo the procedure again.

Our data reinforce this: Although the pain of TP biopsy may be higher, it remains clinically acceptable to almost all patients.

The 30‐day complication rate of 3.4%, chiefly vasovagal episodes immediately after the biopsy, is in keeping with the literature, while infection was noted in 1% of cases. No patient developed urinary retention following the procedure, and importantly, no cases of sepsis were reported. All patients performed pre‐biopsy urine cultures and these were treated accordingly. Patients also received a single dose of cephalexin 500 mg PO post‐biopsy; however, this was no longer routine practice from 2025. A review of 411 consecutive TPPB from this unit found that routine pre‐biopsy urine culture did not alter post‐biopsy infection outcomes in asymptomatic patients, supporting a selective rather than universal approach to pre‐biopsy screening.^25^ The pattern of non‐infectious adverse events, predominantly vasovagal reactions, is consistent with previous reports of TPPB under LA.^29^

For diagnostic yield, clinically significant prostate cancer (ISUP grade group ≥2) was detected in 477 of 769 patients (62.0%), rising across PI‐RADS categories (18.3% at PI‐RADS 1–2, 22.6% at PI‐RADS 3, 70.0% at PI‐RADS 4 and 88.4% at PI‐RADS 5). This overall detection rate is consistent with the pooled csPCa yields reported in contemporary MRI‐directed TP series and meta‐analyses,^18^, ^19^ while the stepwise PI‐RADS gradient mirrors the expected performance of an MRI‐led pathway and accords with the randomised TRANSLATE trial, in which the TP route detected more clinically significant cancer than the transrectal approach.^16^

Cost‐effectiveness considerations extend beyond the choice of anaesthetic for prostate biopsy. In the final analysis of the PREVENT trial (*n* = 742), no infection was recorded after TPPB compared with 1.6% after TRUS biopsy (95% CI −3.5% to −0.3%; *p* = 0.02).^30^ Further economic analysis of the PREVENT trial found office‐based TPPB to be less costly and more effective, saving $63 000 USD per 1000 patients.^31^ These advantages may be driven by the difference of infectious complications between TPPB and TRUS biopsy and avoidance of routine antibiotic prophylaxis. The economic difference is most sensitive to infection rate of TRUS biopsy in this cohort and is correspondingly weaker in services where TR infection rates are already low. In our own service, transitioning from theatre‐based TPPB under general anaesthesia to office‐based TPPB under LA reduced the cost per biopsy from NZ$4200 to NZ$1400.^25^

### 4.3 Anxiety and pain

The moderate positive correlation between patient‐reported anxiety and procedural pain (Spearman ρ = 0.51, 95% bootstrap CI 0.44–0.57) is consistent with literature that links procedural anxiety to procedural pain across surgical and interventional settings and is in keeping with the T‐PAIN observation that older patients—who may on average experience less procedural anxiety—report less pain.^22^ The 10‐fold higher rate of severe pain among patients above the median anxiety score (10.8% vs. 1.0%) is clinically actionable. Of note, the anxiety VAS was captured after the procedure, on the same questionnaire as the pain VAS, so we cannot determine whether pre‐procedural anxiety drove procedural pain or whether recall of anxiety was coloured by the pain actually experienced. Recording a pre‐procedural anxiety score for future research would provide greater insights into this and screen for patients who may further benefit from anxiety‐reducing strategies—pre‐procedural counselling, verbal reassurance, music/distraction and considerations of oral anxiolytics (not used in our centre).

### 4.4 Willingness to repeat

98.9% of cases were willing to undergo a further LA‐TPPB if indicated. The eight patients (1.1%) who would not repeat the procedure differed on pain, anxiety and distress, and two of them had a biopsy that was aborted before completion. 42 of the 45 patients (93%) who reported severe pain still said they would undergo a repeat biopsy; pain alone therefore does not dictate this decision.

### 4.5 Strengths and limitations

The strengths of this study are its large cohort (777 cases) of consecutive patients, the uniform LA regimen utilised and the near‐complete capture of both the pain (97.8%) and the willingness‐to‐repeat (97.3%) outcomes. The integration of anxiety, distress, physical needs, emotional needs and satisfaction into a single questionnaire provides additional context that a pain score alone would not.

Limitations include this study's observational, single‐centre design: Firstly, the case mix, operator practice and the standardised LA regimen are specific to one service and may not be generalisable. Secondly, two operators performed 99% of cases, so operator‐level confounding cannot be excluded. Thirdly, pain was captured at a single post‐procedure timepoint, so we cannot describe the pain trajectory thereafter. Fourthly, anxiety, pain and willingness to repeat were all captured on the same post‐procedure questionnaire, so the associations we report are cross‐sectional rather than predictive. Although procedural duration was recorded contemporaneously by the operating clinician using predefined procedural landmarks, the absence of independent clocked measurement may have introduced some measurement variability. Future prospective studies could enhance precision by using standardised real‐time recording by a designated independent observer. Finally, complications were only noted at index visit.

### 4.6 Implications

For clinical practice, these data support the use of freehand TPPB under LA alone in the outpatient clinic,^27^ with the expectation that approximately 6% of patients will report severe pain and approximately 1% will be unwilling to undergo a repeat procedure. For service design, the anxiety–pain association argues for a brief structured pre‐procedural anxiety screen—even a single VAS in the waiting room—with a defined intervention pathway for patients identified as highly anxious.

Delivering these biopsies in the clinic rather than the operating theatre also released approximately 12 full‐day theatre lists annually in our service, equivalent to a 10% increase in elective theatre capacity.^25^

For research, a randomised comparison of anxiety‐reduction techniques in patients with high pre‐procedural anxiety; prospective collection of step‐stratified pain (probe insertion, skin infiltration, deep block, core sampling) to identify which step contributes most to overall pain; and longitudinal follow‐up of pain and complications.

## 5 CONCLUSION

Freehand TPPB under LA alone, performed in an outpatient urology clinic, is well tolerated: median overall procedural pain is low (VAS 2.0), severe pain is uncommon (5.9%), procedural failure is rare (0.6%), and 98.9% of patients would undergo the procedure again if clinically indicated. Patient‐reported anxiety is moderately associated with procedural pain and represents a plausible modifiable target. These findings support freehand‐LA TPPB as an acceptable default for contemporary prostate cancer diagnosis and make the case for routine integration of patient‐experience measures into the outpatient biopsy pathway.

## AUTHOR CONTRIBUTIONS

**Keu Maoate:** Writing; methodology; conceptualisation. **Josh Tiro:** Writing; investigation. **Ali Hooshyari:** Investigation. **Christian Kirk:** investigation. **Maria McCoy:** Data curation. **Lodewikus Vermeulen:** Methodology; investigation; writing. **Amir Zarrabi:** Writing. **Luiz Gustavo Modelli de Andrade:** Statistical analysis; data curation. **Paulo Roberto Kawano:** Statistical analysis; data curation. **Flavio Vasconcelos Ordones:** Supervision; conceptualisation; investigation; writing.

## FUNDING

This study received no specific funding from any agency in the public, commercial or not‐for‐profit sectors.

## CONFLICT OF INTEREST STATEMENT

The authors declare that they have no conflicts of interest.

## Data Availability

De‐identified analytic data may be made available on reasonable request to the corresponding author, subject to institutional data‐governance approval.
